# New-onset organ dysfunction as a screening tool for the identification of sepsis and outcome prediction in dogs with systemic inflammation

**DOI:** 10.3389/fvets.2024.1369533

**Published:** 2024-04-04

**Authors:** Elena Ciuffoli, Roberta Troìa, Cecilia Bulgarelli, Alessandra Pontiero, Francesca Buzzurra, Massimo Giunti

**Affiliations:** Department of Veterinary Medical Sciences, Alma Mater Studiorum – University of Bologna, Bologna, Italy

**Keywords:** canine, base excess, stupor, coma, acute kidney injury, hemostatic dysfunction

## Abstract

**Introduction:**

Sepsis in people is defined as a life-threatening organ dysfunction (OD) caused by a dysregulated host response to infection. In veterinary medicine, sepsis is still defined by the presence of systemic inflammation plus the evidence of infection. Based on recent veterinary studies, multiorgan dysfunction syndrome (MODS) has been associated with a worse outcome in sepsis. Thus, the screening for OD is warranted to identify the most critically ill patients. The aim of this study was to investigate the diagnostic value of new-onset OD for the prediction of sepsis and outcome in a population of critically ill dogs with systemic inflammation.

**Materials and methods:**

Dogs admitted to the Emergency Room and/or the Intensive Care Unit with systemic inflammation, defined by a serum C-reactive protein concentration > 1.6 mg/dL, were retrospectively included. Enrolled dogs were categorized according to the presence of sepsis or non-infectious systemic inflammation. The presence of newly diagnosed OD was assessed based on criteria adapted from human literature and previously reported canine criteria.

**Results:**

275 dogs were included: 128 had sepsis and 147 had non-infectious systemic inflammation. The frequency of new-onset OD was not different between these groups. Only the presence of fluid-refractory hypotension was significantly associated with a diagnosis of sepsis (OR 10.51, 3.08–35.94; *p* < 0.0001). The frequency of at least two ODs was significantly higher in non-survivors compared to survivors, according to both the human and the veterinary criteria considered for the study (*p* = 0.0001 and *p* = 0.0004, respectively). Specifically, the presence of acute kidney injury, stupor or coma, prolonged Prothrombin Time and decreased Base Excess were associated with a higher risk of death in the multivariate binary logistic regression.

**Discussion:**

In this population of critically ill dogs with systemic inflammation, the detection of newly diagnosed ODs was not able to predict sepsis diagnosis, other than the presence of fluid-refractory hypotension. However, given the strong prognostic significance associated with ODs, our results support the early screening for ODs in any severe inflammatory critical care condition to identify high-risk patients and optimize their management.

## Introduction

Sepsis in people is defined as life-threatening organ dysfunction caused by a dysregulated host response to infection, and is a leading cause of mortality and critical illness worldwide (1). Clinical criteria best identifying infected patients most likely to have sepsis include existing scores of organ dysfunction, as the Sequential [Sepsis-related] Organ Failure Assessment (SOFA) score (2). Currently, in veterinary medicine, sepsis is still defined as a host’s systemic inflammatory response syndrome (SIRS) to infection, and at least two of four SIRS criteria plus a suspected septic focus are required to confirm the diagnosis (3). Despite still based on the latter criteria for diagnosis, sepsis in dogs and cats is frequently complicated by organ dysfunction (OD) (4). Nonetheless, the value of OD for the prediction of sepsis is not known in veterinary medicine (5, 6). Recent studies in septic dogs and cats highlighted that the presence of severe sepsis or Multiorgan Dysfunction Syndrome (MODS) is associated with worse outcomes compared to uncomplicated sepsis, pointing out that the screening for OD is advocated in order to identify the most critically ill patients (5, 6).

The aims of the current study were: (1) to investigate the value of new-onset OD for the prediction of sepsis in a population of critically ill dogs with systemic inflammation admitted at the Emergency Room (ER) and/or the Intensive Care Unit (ICU); (2) to assess its prognostic potential for outcome prediction in the same population.

We hypothesize that new-onset of OD in critically ill dogs with systemic inflammation is associated with a diagnosis of sepsis, and that the number and types of dysfunctional organ systems has prognostic value in this setting.

## Materials and methods

### Inclusion criteria and data collection

Dogs examined at the Veterinary University Hospital (VUH) between January 2017 and December 2020 were retrospectively included. To be eligible for inclusion, dogs had to fulfill the following criteria: (1) be admitted at the ER and/or the ICU; (2) had evidence of systemic inflammation defined as a serum C-reactive Protein (CRP) > 1.6 mg/dL, as previously reported (7); (3) undergone complete clinical and clinicopathological evaluation for the assessment of OD; (4) had a clear final diagnosis. Based on their final diagnosis, enrolled dogs were divided in two groups: dogs with sepsis and dogs with non-infectious systemic inflammation. Sepsis diagnosis was achieved combining the presence of systemic inflammation with evidence of a septic focus, confirmed by cytology, microbiology, histopathology, or real-time polymerase chain reaction. Clinical records of enrolled dogs were reviewed, and the following variables at the time of ER or ICU admission were recorded: signalment, body weight, clinical findings including rectal temperature, heart rate, respiratory rate, mental status (classified as: normal mentation, depressed mentation, stupor, coma), non-invasive systolic blood pressure measurement (petMAP Graphic, Ramsey Medical, Sydney, Australia; Minidop ES-100 VX, Hadeco, Kawasaki, Japan) and the need to receive oxygen therapy. The SIRS criteria were evaluated for all included dogs: rectal temperature (<38°C, >39°C), heart rate (>120 beats per minute), respiratory rate (>20 breaths per minute), white blood cell (WBC) count (<6 ×10^3^/mm^3^, >16 ×10^3^/mm^3^) with percent bands (>3%). SIRS was deemed present if dogs had at least 2/4 criteria (8). Regarding clinicopathological screening, all analyses were performed at the clinical pathology laboratory of our VUH, and samples were obtained according to standard operating procedures. The following clinicopathological data were recorded: blood gas, electrolytes and lactate measurements (ABL800 FLEX, Radiometer Medical ApS, Denmark); complete blood count (ADVIA 2120, Siemens Healthcare Diagnostics, Tarrytown, NY); serum chemistry including CRP concentration (Olympus AU 480, Olympus/Beckman Coulter, Brea; CA); coagulation assays (SEAC Clot 2S and BTF II Siemens, Marburg, Germany). Data collected were used to assess disease severity by calculating the canine Acute Patient Physiologic and Laboratory Evaluation fast (APPLE_fast_) score, 5-variable score contained glucose (mg/dL), albumin (g/dL), lactate (mg/dL), platelet count (x10^9^/L), mentation score (9). The study was approved by the local Institutional Animal Care and Use Committee (protocol number ID 846).

### Organ dysfunction and outcome

At the time of hospital admission, the presence of newly diagnosed ODs was assessed considering two different diagnostic criteria: (1) those adapted from a human study conducted in septic people published from Swenson et al. in 2018 (10); (2) those reported in the available canine literature to address MODS (11) (Table 1). The number of dysfunctional organs according to both the human and canine criteria were also recorded. MODS was defined by the presence of at least two dysfunctional organs, simultaneously. Outcome was defined as survival to hospital discharge, death, or euthanasia for disease severity and perceived guarded prognosis.

**Table 1:** Criteria adapted from human and veterinary literature for the identification of organ dysfunction: Swenson et al. (10), Troia et al. (11).

Organ Dysfunction	Human criteria (Swenson et al. (10))	Veterinary criteria (Troia et al. (11))
Renal	Creatinine >2.0 mg/dL	Creatinine >1.4 mg/dL or oliguria
Respiratory	Sp $O_{2}$ < 95%	Sp $O_{2}$ < 95%
Hepatic	Total bilirubin >0.4 mg/dL	Total bilirubin >0.4 mg/dL
Cardiovascular	Fluid-refractory hypotension requiring vasopressor support	Fluid-refractory hypotension requiring vasopressor support
Hemostatic	PT > 9 s or aPTT >16 s or platelet count <160.000/mm^3^	PT > 7.5 s or aPTT >16.5 s or platelet count <160.000/mm^3^
Neurologic	Presence of stupor or coma	Not applied
Unexplained Acidemia	pH < 7.34 HC $O_{3}^{-}$ < 21 mmol/L Base Excess < −3 mmol/L	Not applied
Hyperlactatemia	Serum lactate >2.0 mmol/L	Not applied

aPTT, activated partial thromboplastin time; PT, prothrombin time.

### Statistical analysis

Statistical analysis was performed using a statistical software package (MedCalc Statistical Software version 19.1.3, Ostend, Belgium 2019). Data were assessed for normality with the D’Agostino-Pearson test and evaluated by descriptive statistics. The Mann–Whitney U-test was used to compare continuous variables between groups. Categorical variables were compared using the Fisher’s exact test. Univariate and multivariate logistic regressions were used to identify variables able to predict diagnosis of sepsis and outcome; results were presented as odds ratio (OR) and 95% confidence interval (CI). Significance was set at *p* < 0.05.

## Results

A total of 403 medical records of critically ill dogs with a CRP > 1.6 mg/dL, admitted at the ER or ICU in the study period were reviewed. Of these, 128 patients were excluded for the lack of complete clinicopathological data or unclear final diagnosis. The final study population included 275 dogs: 128 (47%) had sepsis and 147 (53%) had non-infectious systemic inflammation. In the overall population there were 83/275 (30%) spayed females, 78/275 (28%) intact females, 30/275 (11%) neutered males and 84/275 (31%) intact males. Many breeds were represented, with the most common being Mixed breed (*n* = 82), Labrador retriever (*n* = 11) and German Shepherd (*n* = 11), Golden Retriever (*n* = 9), English Setter (*n* = 9), Miniature Poodle (*n* = 8), and Maltese (*n* = 8). Median age was 9 years (range 0.1–19), median body weight was 30.1 kg (range 0.4–59.8). Underlying causes for sepsis included reproductive tract infections (27 pyometra, 4 metritis; *n* = 31), intra-abdominal infections (17 septic peritonitis, 1 penetrating abdominal trauma; *n* = 18), respiratory tract infections (14 pneumonia, 1 pulmonary abscess, 3 pyothorax; *n* = 18), complicated urinary tract infections (12 pyelonephritis, 4 prostatic abscess and prostatitis; *n* = 16), skin and subcutaneous tissue infections (8 bite wounds, 5 necrotizing fasciitis, 1 subcutaneous abscess; *n* = 14), parvoviral enteritis (*n* = 13), miscellaneous diseases (6 bacteremia during severe acute hemorrhagic gastroenteritis, 4 leptospirosis, 4 chemotherapy-induced febrile neutropenia, 1 diabetic ketoacidosis complicated with septic shock, 1 rectal laceration, 1 bacterial cholangitis, 1 endocarditis; *n* = 18). Underlying causes for non-infectious systemic inflammation included immune mediated conditions (21 immune mediated hemolytic anemia, 5 immune mediated thrombocytopenia; *n* = 26), neoplasia (*n* = 22), cardio-pulmonary diseases (18 cardiogenic pulmonary edema, 3 pulmonary thromboembolism, *n* = 21), metabolic diseases such as acute on chronic kidney injury (*n* = 17), diabetic ketoacidosis (*n* = 9), seizures (*n* = 9), enterectomy for intestinal foreign body (*n* = 7), miscellaneous diseases 8 acute gastroenteritis, 4 gastric dilatation volvulus, 4 snake envenomation, 3 intestinal intussusception, 3 heat stroke, 2 pancreatitis, 2 cholangitis, 1 metaphyseal osteopathy, 1 intoxication by anticoagulant rodenticides, 1 exertional rhabdomyolysis, 1 blunt trauma, 1 hepatic lobe torsion, 1 acute kidney injury caused by non-steroidal anti-inflammatory drug (NSAID), 1 anaphylaxis, 1 Addison’s disease, 1 perineal hernia, 1 traumatic diaphragmatic hernia; (*n* = 36).

Main clinical and clinicopathologic findings for dogs with sepsis and non-infectious systemic inflammation are reported in Table 2.

**Table 2:** Descriptive statistics of selected clinical and clinicopathological variables in dogs with sepsis and dogs with non-infectious systemic inflammation (NSI).

Variable	RI	Sepsis	*n*	NSI	*n*	*p* value
Clinical data						
Body temperature (°C)	38–39	39.0 (33.0–41.7)	128	38.6 (34.0–41.7)	147	**0.0004**
Heart rate (bpm)	60–120	133 (52–260)	128	140 (60–230)	147	0.7766
Respiratory rate (rpm)	10–40	40 (16–120)	116	44 (10–120)	123	0.1689
SBP (mmHg)	120–170	133 (40–199)	93	145 (80–250)	98	**0.0051**
Blood gas analysis						
pH	7.34–7.40	7.35 (7.09–7.55)	121	7.31 (6.90–7.52)	129	**0.0019**
HCO_3_ (mmol/L)	18.0–23.2	18.7 (± 4.5)	121	17.5 (3.9–40.1)	129	**0.0191**
Base Excess (mmol/L)	−2.0 – 2.5	−4.8 (−29.0–5.5)	121	−6.6 (−28.0–16.8)	129	**0.0153**
Lactate (mmol/L)	0.5–2.0	2.3 (0.4–9.9)	122	2.6 (0.4–13.2)	134	0.2060
Hematology						
WBCs (x $10^{9}$ /L)	6–17	16.37 (0.90–124.40)	126	16.70 (3.27–147.10)	146	0.3390
Platelets (x $10^{9}$ /L)	160–500	235.0 (8.0–729.0)	126	257.0 (3.0–998.0)	146	0.5404
Chemistry						
Glucose (mg/dL)	65–115	101 (20–665)	128	117 (52–1,126)	147	**< 0.0001**
Creatinine (mg/dL)	0.75–1.40	1.00 (0.28–14.80)	128	1.09 (0.33–18.54)	147	0.1474
Total bilirubin (mg/dL)	0.07–0.33	0.25 (0.01–107.00)	128	0.23 (0.01–20.93)	147	0.7869
Albumin (mg/dL)	2.75–3.85	2.40 (± 0.56)	128	2.75 (± 0.52)	147	**< 0.0001**
CRP (mg/dL)	0.00–0.85	20.90 (1.74–61.92)	128	7.03 (1.63–56.20)	147	**<0.0001**
Coagulation						
PT (sec)	5.0–7.5	7.1 (5.1–35.0)	93	7.0 (5.0–15.7)	79	0.9559
aPTT (sec)	8.0–16.5	13.8 (7.5–81.7)	93	12.9 (8.0–32.2)	79	0.2207

Data are reported as mean ± standard deviation or median and range (minimum-maximum value), based on their distribution.aPTT, activated partial thromboplastin time; CRP, C – Reactive Protein; n, number of dogs; PT, prothrombin time; RI, reference interval; SBP, systolic blood pressure; WBCs, white blood cells. Bold characters refer to a *p* value < 0.05.

In the overall population of critically ill dogs, 94 (34%) dogs died, and 181 (66%) survived. The frequency of death was similar between dogs with sepsis and those with non-infectious systemic inflammation (34% vs. 34%, *p* = 1.000).

According to published criteria, the frequency of SIRS was similar between dogs with and without sepsis: 119 (93%) of septic dogs and 128 (87%) dogs with non-infectious systemic inflammation (*p* = 0.11).

### Association between OD and diagnosis of sepsis

In the overall population of critically ill dogs, the number of ODs was distributed as follows: acidemia (*n* = 202), hyperlactatemia (*n* = 152), acute kidney injury (*n* = 96), hyperbilirubinemia (*n* = 68), prolonged coagulation times (either activated partial thromboplastin time, aPTT, or prothrombin time, PT) (*n* = 68), thrombocytopenia (*n* = 64), hypoxemia (*n* = 41), fluid-refractory hypotension (*n* = 26), stupor or coma (*n* = 18).

Overall, the frequency of new-onset ODs was not different between dogs with sepsis and those with non-infectious systemic inflammation. However, based on the univariate logistic regression analysis, the frequency of fluid-refractory hypotension was significantly associated with a diagnosis of sepsis (OR 10.51, 3.08–35.94; *p* < 0.0001).

No difference in the number of ODs was reported between dogs with sepsis and dogs with non-infectious systemic inflammation. In particular, according to the veterinary criteria, 45/128 (35%) dogs with sepsis and 50/147 (34%) dogs with non-infectious systemic inflammation had at least two or more ODs (*p* = 0.899).

### Association between OD and outcome

According to the human criteria, the frequency of at least 2 ODs was significantly higher in non-survivors compared to survivors (88% vs. 68%, *p* = 0.0001). Similarly, when the veterinary criteria were considered, the frequency of at least 2 ODs was significantly higher in non-survivors compared to survivors (49% vs. 27%, *p* = 0.0004).

The presence of acute kidney injury, stupor or coma, hyperbilirubinemia, hyperlactatemia, fluid-refractory hypotension, prolonged PT and decreased Base Excess (BE) as a marker of acidemia were independently associated with a higher risk of death by univariate logistic regression analysis. When the multivariate binary logistic regression analysis was performed, the presence of acute kidney injury, stupor or coma, prolonged PT and decreased BE were the variables retained by the model (Table 3).

**Table 3:** Significant variables based on univariate and multivariate analysis for outcome prediction.

Outcome				
Variable					RC	SEM	OR (95% CI)	*p*
Univariate logistic regression
Acute kidney injury	1.42	0.27	4.15 (2.44–7.04)	< 0.0001
Stupor/coma	1.73	0.54	5.65 (1.95–16.38)	0.0006
Hyperbilirubinemia	0.70	0.29	2.01 (1.14–3.53)	0.0158
Hyperlactatemia	0.20	0.06	1.22 (1.09–1.38)	0.0006
Fluid-refractory hypotension	1.63	0.45	5.12 (2.13–12.29)	0.0001
Prolonged Prothrombin Time	0.23	0.09	1.26 (1.07–1.49)	0.0014
Decreased Base Excess	−0.12	0.02	0.89 (0.84–0.93)	< 0.0001
Multivariate logistic regression				
Acute kidney injury	1.38	0.43	3.97 (1.71–9.24)	0.0014
Stupor/coma	2.13	0.94	8.43 (1.33–53.25)	0.0234
Prolonged Prothrombin Time	0.18	0.09	1.20 (1.00–1.44)	0.0437
Decreased Base Excess	−0.07	0.03	0.93 (0.87–0.99)	0.0472

CI, Confidence interval; OR, Odds Ratio; RC, Repeatability Coefficient; SEM, Standard Error of the Mean.

## Discussion

In our population of critically ill dogs, presence of new-onset OD was not related to a diagnosis of sepsis. Indeed, only fluid-refractory hypotension was specifically linked to the presence of sepsis. Thus, the presence of fluid-refractory shock has to spur clinicians to screen for sepsis and treat patients according to the sepsis bundle, even if the septic focus is not obvious or remains undetected (1).

Sepsis definition has changed over the last few decades. The first proposed definition and diagnosis of sepsis was based on the SIRS criteria, which is referred to as Sepsis-1 (12, 13). Because of the low specificity of the SIRS criteria and due to the common occurrence of sepsis-induced OD that is strongly related to worse outcomes, the current definition of sepsis in humans emphasizes the presence of organ system failure or dysfunction. Indeed, according to the new consensus definition Sepsis-3, sepsis is now defined as the life-threatening organ dysfunction caused by a dysregulated host response to infection (1).

In veterinary medicine, sepsis definition is still based on the SIRS criteria, and eventually on the presence of systemic inflammation detected by a rise in major acute phase proteins, like CRP in dogs (7, 14). For this reason, we enrolled critically ill dogs with a serum CRP at admission in ER above 1.6 mg/dL, as previously suggested (7), with the purpose of evaluating the diagnostic value of new-onset OD for sepsis diagnosis, as well as its prognostic potential.

None of the additional ODs considered for this study, based on both the human and veterinary criteria used, was able to identify sepsis in our population. There are multiple possible explanations that could be hypothesized behind this result, and partially linked to our study design: first of all, enrolled dogs were all characterized by the presence of systemic inflammation with a high frequency of OD. It is well known that OD is not unique for patients with sepsis, as many non-infectious critical care conditions, such as trauma and pancreatitis, are indeed accompanied by the acute onset of organ failure (13, 15). According to the retrospective nature of our study, we were not able to record potential septic complications developed during hospitalization in the latter patients. Similarly, the retrospective identification of septic dogs poses a significant challenge in the enrollment of our critically ill dogs, as the final diagnosis of sepsis could have been questionable in some cases and, on the other hand, might not have been definitely excluded in some patients classified with non-infectious systemic inflammation.

The new onset of specific ODs was able to predict outcome in our population of critically ill dogs admitted to ER and/or ICU. Specifically, patients with acute kidney injury, presence of stupor or coma, a prolonged Prothrombin time and an unexplained acidemia, showed higher risk of non-survival. Acute kidney injury in ICU is a heterogeneous syndrome with regard to exposure (eg, sepsis, surgery, toxicity), pathophysiology (eg, inflammation, hypoperfusion) and clinical presentation. The prognostic importance of AKI has been well demonstrated in critically ill humans, acting both as an indicator of disease severity and an independent predictor of mortality (16, 17). Due to the retrospective design of our study, we were not able to further characterize AKI etiologies and phenotypes (transient vs. intrinsic AKI), as previously suggested (18). Nonetheless, the results of the present study are in line with previous findings in dogs, overall corroborating the need for AKI screening in critically ill patients (18). With regard to neurological dysfunction, this appears to be one of the most important risk factors for sepsis-related mortality in humans, although only a low prevalence is normally reported (19). Neurological dysfunction is poorly defined in small animals, being not even considered in some of the available criteria for MODS in dogs (11, 20). Our results highlight the prognostic significance of the presence of stupor and coma in the current study population, underlining the need for a standardized neurological assessment in critically ill dogs. Coagulopathy in the course of critical care diseases results from interactions between inflammation, endotheliopathy and coagulation leading to microthrombi formation, consumption of coagulation factors, and finally in the development of disseminated intravascular coagulation (21). A reduced activity of endogenous anticoagulants and a possible imbalance of homeostasis with a tendency to a procoagulant state has been demonstrated in dogs with sepsis (22). Furthermore, selected hemostatic variables acted as predictors of survival in critically ill dogs (5, 23). Although our results do not allow for characterization of the phenotype of the hemostatic derangement, these results corroborate its prognostic significance in critical dogs. Finally, also the presence of unexplained acidemia was associated with a worse outcome in our study. In septic people, both lactate and base deficit have been proposed as endpoints for resuscitation and outcome predictors (24). Data in veterinary literature are limited to selected studies pointing out the prognostic role of acidemia and base deficit in critically ill dogs with sepsis and different emergency conditions (25, 26).

Overall, our results support the association between the development of OD and a poor prognosis (11). Therefore, given the lack of specific treatment for MODS, the early recognition of ODs is imperative to identify the most critical ICU subpopulation and to improve its intensive management, aiming to ameliorate outcomes.

The present study has several limitations. Due to the retrospective design, the diagnostic and therapeutic protocol of patients included was not standardized, but chosen by attending clinicians, and for this reason some clinicopathological data were missing for some cases, potentially affecting a complete evaluation of organ dysfunction. In addition, despite the inclusion of critically ill dogs with a clear final diagnosis, diagnosing sepsis is always a challenge: potential septic patients might have been erroneously diagnosed as non-septic, and at the same time, dogs that were not septic at the time of ER/ICU arrival could have then developed sepsis during hospitalization, causing unpredictable overlap in the two subpopulations considered in the study, as previously discussed. Finally, for some of the enrolled dogs there were no baseline laboratory data; hence, the diagnosis of the new-onset OD based on laboratory criteria (eg, acute kidney injury, hyperbilirubinemia) could have been potentially confounded by the possibility of previous disease or comorbidity.

## Conclusion

To conclude, our results support the importance of the early identification of ODs in the veterinary critical care setting, and highlight the prognostic role of specific ODs like neurological, renal, hemostatic dysfunction and unexplained acidemia in dogs, early at the time of ER or ICU arrival. In this population, the detection of ODs was not helpful for diagnosing sepsis, and only the finding of fluid-refractory hypotension was clearly associated with sepsis. However, searching for a septic process in a patient showing new-onset ODs has always to be pursued. Finally, the value of the current results has to be investigated in larger population of critically ill patients, ideally considering a multi-centric prospective study design.

## Data availability statement

The raw data supporting the conclusions of this article will be made available by the authors, without undue reservation.

## Ethics statement

The study was approved by the local Institutional Animal Care and Use Committee (protocol number ID 846). The studies were conducted in accordance with the local legislation and institutional requirements. Written informed consent was obtained from the owners for the participation of their animals in this study.

## Author contributions

EC: Writing – original draft, Writing – review & editing. RT: Writing – original draft, Writing – review & editing. CB: Writing – review & editing. AP: Writing – review & editing. FB: Writing – review & editing. MG: Writing – original draft, Writing – review & editing.
